# The current role of secondary cytoreductive surgery for recurrent ovarian cancer

**DOI:** 10.3389/fonc.2022.1029976

**Published:** 2022-10-21

**Authors:** Eelco de Bree, Dimosthenis Michelakis, Elisavet Anagnostopoulou

**Affiliations:** Department of Surgical Oncology, Medical School of Crete University Hospital, Heraklion, Greece

**Keywords:** ovarian cancer recurrence, secondary cytoreductive surgery, patient selection, platinum-sensitive, predictive tools

## Abstract

Ovarian cancer represents worldwide the second most frequent and the most fatal gynecological malignancy, with approximately two thirds of the patients presenting with advanced disease. Cytoreductive surgery, primary or after neoadjuvant chemotherapy, in combination with platinum-based chemotherapy is the standard of care for these patients. Despite the improvement in quality of cytoreductive surgery as well as development of novel drugs and chemotherapy regimens, still most women with ovarian cancer will ultimately develop recurrent disease and die of their disease. In contrast to the management of primary disease, the standard treatment of patients with recurrent ovarian cancer remains a topic of debate. While platinum-based or second line systemic chemotherapy, depending on the time after last platinum treatment, is standard of care, the role of secondary cytoreductive surgery has been a controversial issue for the last decades. Potential outcome benefit must be also weighed against the risk of severe surgical morbidity, impairment of quality of life and costs. In platinum-resistant recurrent disease, i.e., relapse after less than 6 months from the last platinum-based chemotherapy for primary disease, secondary cytoreduction seems generally not to be indicated due to its aggressive biological behavior and the absence of effective systemic treatment. In this comprehensive review, the current role of cytoreductive surgery in platinum-sensitive recurrent ovarian cancer is discussed thoroughly in view of the results of most recent randomized trials and a meta-analysis. There seems to be definitely a role for secondary cytoreductive surgery in selected patients with ovarian cancer recurrence in whom complete resection of macroscopic disease is feasible. However, its role should be continuously reviewed due to the changing systemic treatment of patients with ovarian cancer recurrence over time.

## Introduction

In recent global cancer statistics, ovarian cancer represents the third most frequent gynecological malignancy and the second cause of death from gynecological cancer (1). It has been estimated that in 2022 almost 20.000 women will be newly diagnosed with ovarian cancer and almost 13.000 will die from this disease in the U.S.A (2). The vast majority of ovarian cancer patients have already advanced disease with peritoneal metastases at diagnosis (2). The treatment of choice for primary advanced ovarian cancer has been the combination of primary (or interval) cytoreductive surgery (CRS), aiming for complete resection of all visible disease, and systemic chemotherapy (3, 4). Whereas the standard chemotherapy has been the combination of carboplatin and paclitaxel (3, 4), more recent studies have demonstrated an increase of progression-free survival by additional systemic treatment with bevacizumab or a PARP inhibitor (5–11). In meta-analyses (12, 13), primary complete CRS, without macroscopic residual disease, has been associated with a significant survival benefit. Outcome after incomplete primary CRS was substantially inferior. The theoretical benefit from CRS relates to removing large tumor volumes that have a decreased growth fraction and poor blood supply, thereby improving the efficacy of chemotherapeutic agents. Additionally, CRS is believed to remove chemo-resistant clones of cancer cells by eradicating as much as possible tumor masses and to enhance host immunological response. Complete CRS may circumvent acquired drug resistance after adjuvant chemotherapy (14, 15). Despite the improvement of the quality of primary CRS and the development of new systemic treatment regimens, resulting in a high percentage of clinical remission after completion of initial treatment, approximately 80% of the women with advanced epithelial ovarian cancer will ultimately develop recurrence (1, 16, 17). Only 15% of patients with early ovarian cancer experience recurrent disease (18). The standard of care in recurrent ovarian cancer has mainly consisted of systemic treatment, with eventually palliative surgery for complications as bowel obstruction, whereas the role of CRS in this setting has not been well defined yet. In this comprehensive review the current role of secondary CRS in patients with recurrent ovarian cancer will be discussed, especially in view of data of recent randomized controlled studies.

## Secondary cytoreductive surgery

In view of the widespread adoption of primary CRS, it is not unexpected that secondary CRS is strongly considered for patients with recurrent ovarian cancer. This is particular the case for patients with potentially platinum-sensitive disease (i.e., those with recurrence at least 6 months after the last platinum containing therapy) and patients with relatively limited-volume recurrent disease. Platinum-resistant disease represents aggressive biological behavior and in absence of effective systemic treatment secondary CRS is generally considered not to be beneficial. In the past, several retrospective studies and meta-analyses have demonstrated a benefit from secondary CRS, most obviously for patients with platinum-sensitive recurrence and when macroscopic residual disease is very small (optimal CRS) or absent (complete CRS) (17, 19–22). Among all studies, the definition of optimal CRS varies widely from residual disease smaller than 0.25 cm to residual tumor up to 2.5 cm. In an earlier meta-analysis (19), the weighed mean proportion of patients undergoing complete and optimal secondary CRS was 52.2% and 70.3%. In multivariate analysis, the only statistically significant clinical variable independently associated with post-recurrence survival time was the proportion of patients undergoing complete secondary CRS (p=0.019) (19). After controlling for confounding variables, each 10% increase in the proportion of patients undergoing complete CRS was associated with a 3.0 month increase in median cohort overall survival time. The impact of optimal CRS on survival was less obvious. Moreover, in another previous systematic review and meta-analysis (23), overall survival was higher after complete than after optimal CRS, whereas larger residual disease was associated with poorer outcome. The difference in impact on survival between complete and optimal secondary CRS may be caused by the fact that residual disease drives an early development of drug resistance or that recurrent disease that cannot be complete resected, even by an expert team, represent an aggressive tumor biology that can cannot be altered by surgery.

In selected patients, laparoscopic CRS appears to be a feasible and safe approach to complete removal of recurrent ovarian cancer (24). In the case of isolated lymph node recurrence, salvage lymphadenectomy as secondary CRS seems beneficial with a median progression-free survival of 27 months, especially when the platinum-free is longer and the number of involved lymph nodes low, but independently of BRCA mutational status (25). In selected patients, salvage lymphadenectomy may be also performed in a minimal invasive manner (26, 27). Even when recurrent disease involves major vascular structures, vascular procedures can be safely performed with a proper pre-operative planning and may not be an impediment to major gynecological oncological surgery (28).

## Randomized trials

Despite the encouraging results of retrospective studies and meta-analyses, a patient selection bias might have been considerable in these studies and consequently randomized studies are warranted. Moreover, in the era of bevacizumab and PARP inhibitors, which addition to systemic chemotherapy appear to improve progression-free survival significantly among patients responding to salvage treatment for platinum-sensitive relapse (29), the role of secondary CRS may have to be redefined. Recently, five randomized trials were initiated to assess the role of secondary CRS in recurrent ovarian cancer. Unfortunately, two of them, the Dutch SOCCER trial and the EORTC 55963 trial, were prematurely closed due to low recruitment. The most recently published results of the remaining three randomized trials will be discussed below (**Table 1**).

**Table 1:** Results of randomized controlled trials on secondary cytoreductive surgery for recurrent ovarian cancer.

Study	Year	Sec. CRS	N	Selection criteria	Complete CRS	PFS*(months)	HR, p-value	OS*(months)	HR, p-value	Survival for completevs. incomplete CRS*
GOG-0213 (30)	2019	Yes	240	Clinical opinion	67%	18.9	HR=0.82	50.6	HR=1.29	PFS 22 vs. 13 months, HR=0.51
		No	245			16.2	NS	64.7	p=0.08	OS 56 vs. 38 months, HR=0.61
SOC-1 (31)	2021	Yes	182	Tian/iMODEL	77%	17.4	HR=0.58	58.1**	HR=0.82	PFS 19 vs. 13 months
		No	175	score		11.9	p<0.001	53.9	NS	OS >72 (NR) vs. 35 months
DESKTOP III (32)	2021	Yes	206	AGO score	75.5%	18.4	HR=0.66	53.7	HR=0.75	PFS 21 vs. 12 months
		No	201			14.0	p<0.001	46.0	p=0.02	OS 62 vs. 28 months

Sec, secondary; CRS, cytoreductive surgery; N, number of patients; PFS, progression-free survival. OS, overall survival, * median values, ** interim analysis, NR, not reached; HR, hazard ratio.

### The GOG-0213 trial

The Gynecological Oncology Group (GOG) performed the multinational multicenter GOG-0213 trial to assess the role of bevacizumab in recurrent ovarian cancer and whether secondary CRS would increase overall survival among ovarian cancer patients with platinum-sensitive relapse and who were potential surgical candidates (30). Patients with platinum-sensitive recurrent epithelial ovarian cancer considered to be amenable to complete CRS by the surgeon were enrolled in the study. The patients should have had a complete clinical response after the initial treatment and recurrent disease should have been diagnosed at least 6 months after the last chemotherapy. Patients who were not medical fit for major surgery and those with diffuse carcinomatosis, ascites or extra-abdominal disease were excluded. No other specific selection criteria were used. In a 10-year period, 485 patients were randomly assigned to secondary CRS followed by systemic treatment (240 patients) or systemic treatment only (245 patients). Systemic treatment consisted of paclitaxel-carboplatin or gemcitabine-carboplatin. As part of the chemotherapy component of the randomized trial all patients were randomized to the addition of bevacizumab or not to the chemotherapy regimen.

Two hundred twenty five of the 240 patients assigned to surgery actually underwent CRS. In 67% of the cases complete CRS was achieved. The median estimated blood loss was 200 ml and blood transfusion only necessary in 8% of the patients. Bowel resection was performed in 28%, a stoma was created in 2% and the procedure was aborted in 4% of the cases. The 30-day surgery related morbidity was only 9% and the 30-day mortality only 0.4%, whereas no patient underwent repeat laparotomy for complications. Patients in the CRS group experienced a significant decrease in quality of life immediately after surgery. However, after recovery from surgery, there was no difference in quality of life between both groups at time points up to 12 months.

After a median follow-up period of 48.1 months, no significant differences in outcome between both groups were observed. The median overall survival, counted from the time of randomization, was 50.6 months and 64.7 months for the CRS group and no surgery group, respectively (adjusted hazard ratio [HR] 1.29, 95% confidence interval [CI] 0.97-1.72, p=0.08), whereas the progression-free survival was 18.9 months and 16.2 months, respectively (HR 0.82, 95% CI 0.66-1.01). The 3-year overall survival rates were 67% and 74% and the 3-year progression-free survival rates 29% and 20%, respectively. In subgroup analysis, no patient and treatment variables could be identified that were associated with improved overall survival following secondary CRS. In the small group of patients (n=77, 15,9% of the patients) that did not receive bevacizumab after randomization, patients who underwent secondary CRS (n=38, 15.8% of the patients) experienced worse overall survival than those treated by chemotherapy only (n=39, 15,9% of the patients. In the CRS group, complete CRS, when compared with incomplete CRS, was associated with longer overall (HR 0.61, 95% CI 0.40-0.93, median 56.0 vs. 37.8 months) and progression-free survival (HR 0.51, 95% CI 0.36-0.71, median 22.4 vs. 13.1 months). Although patients with complete CRS did not experience an improved overall survival when compared with those who did not undergo surgery (HR 1.03, 95% CI 0.74-1.46, median 56.0 vs. 64.7 months), a benefit regarding progression-free survival was observed after complete CRS (HR 0.62, 95% CI 0.48-0.80, median 22.4 vs. 16.2 months).

### The SOC-1 trial

The Chinese multicenter SOC-1 trial (31) investigated the same hypothesis, i.e. whether secondary CRS is of benefit in platinum-sensitive ovarian cancer recurrence that is potentially completely resectable. Completeness of resectability was predicted by the Tian score, or otherwise called iMODEL score, and PET-CT imaging. The Tian score uses six variables, including FIGO stage, residual disease after primary surgery, platinum-free interval, ECOG performance status, serum level of CA-125 and presence of ascites at recurrence (**Table 2**). A value of ≤ 4.7 is considered to predict a potentially complete CRS (33). Patients with a higher score and a CA-125 >105 U/mL could be included when the principal investigator deemed the disease completely resectable at PET-CT. In a 7-year period, 357 patients were randomized to secondary CRS and systemic chemotherapy (182 patients) or systemic chemotherapy only (175 patients). The chemotherapy regimen consisted of paclitaxel or docetaxel combined carboplatin. Maintenance treatment with bevacizumab or PARP inhibitors was allowed. Patients were excluded when complete CRS was deemed impossible according to the Tian score and PET-CT, in case of re-recurrence, when the patient had received more than first-line chemotherapy only and when comorbidity did not allow major surgery or chemotherapy. Patients were stratified according to participation center, Tian score, completeness of primary CRS and enrollment in the SUNNY study (primary versus interval CRS for primary disease).

**Table 2:** Tian or iMODEL score system. Score ≤4.7 represents low-risk and score > 4.7 high-risk for not achieving complete secondary CRS (33).

Impact factors			Scoring			
	0	0.8	1.5	1.8	2.4	3.0
FIGO stage	I/II	III/IV				
Residual disease after primary CRS	0		>0			
Progression-free interval (months)	≥16				<16	
ECOG performance status	0-1				2-3	
Ca-125 level at recurrence (U/mL)	≤105			>105		
Ascites at recurrence	absent					present

FIGO, Fédération Internationale de Gynécologie et d’Obstétrique; CRS, cytoreductive surgery; ECOG, Eastern Cooperative Oncology Group.

In 77% of the patients, secondary CRS was considered complete, with no gross residual disease. Five percent of the patients who underwent secondary CRS experienced grade 3-4 30-day surgical morbidity, while no patient had died at 60 days in either group. After a median follow-up of 36.0 months, median progression-free survival, counted from the day of randomization, was 17.4 months in the secondary CRS group and 11.9 months in the chemotherapy only group (HR 0.58, 95% CI 0.45-0.74, p<0.0001). In subgroup analysis, the statistically significant progression-free survival benefit of secondary CRS remained in almost all subgroups and in none of the subgroups the outcome was worse after secondary CRS. Whereas complete CRS was associated with better progression-free survival than chemotherapy only (HR 0.50, 95% CI 0.37-0.66), incomplete CRS and chemotherapy only displayed similar progression-free survival curves (HR 0.91, 95% CI 0.61-1.36). While the investigators planned to assess definite overall survival outcome after further maturation of data, a prespecified interim overall survival analysis showed no statistically significant difference between both groups, with a median overall survival of 58.1 and 53.9 months, respectively (HR 0.82, 95% CI 0.57-1.19). However, patients with complete CRS experienced a better overall survival (HR 0.59, 95% CI 0.38-0.91) and patients with incomplete CRS a worse overall survival than patients who received chemotherapy only (HR 1.79, 95% CI 1.07-2.99). Time intervals to first and second subsequent systemic treatment, key endpoints between progression-free and overall survival, were also longer in the secondary CRS and chemotherapy group when compared with the chemotherapy only group. From the 130 patients in the chemotherapy only group who had a subsequent relapse, 48 (37%) underwent surgery. Assessment of quality of life did not show differences among both groups of patients.

### The DESKTOP III trial

In the third international multicenter randomized study, the DESKOP III trial (32), 407 ovarian cancer patients with a first platinum-sensitive relapse (i.e., with an interval of at least 6 months without platinum-based chemotherapy) and a positive AGO score, to assure a high likelihood of complete secondary CRS, were allocated to undergo secondary CRS and subsequently to receive platinum-based chemotherapy (206 patients) or to receive platinum-based chemotherapy alone (201 patients). A patient with a positive AGO score should have platinum-sensitive relapse, an ECOG performance status of 0, ascites of less than 500 ml and complete primary CRS at initial treatment (34) (**Table 3**).

**Table 3:** AGO score.

Predictive parameters for complete secondary cytoreductive surgery
Platinum-sensitive recurrent ovarian cancer (interval of ≥6 months without platinum-based chemotherapy
ECOG performance status of 0
No residual disease after primary surgery (or, alternatively if information not available, FIGO I/II)
Ascites of less than 500 ml at preoperative imaging

FIGO, Fédération Internationale de Gynécologie et d’Obstétrique; ECOG, Eastern Cooperative Oncology Group. The AGO score is positive when all parameters are encountered (22, 34).

Complete secondary CRS was achieved in 76% of the patients who underwent surgery. The median operation time was 222 minutes, raging from 150 to 300 minutes. Bowel resection was performed in 36%, a stoma was created in 8% and partial hepatectomy was performed in 5% of the patients. The median estimated blood loss was 250 ml and blood transfusion only necessary in 17% of the patients. No perioperative death was recorded. Reoperation for complications had to be performed in 3.7% of the patients. The majority of patients in both groups received at least five cycles of platinum-based chemotherapy postoperatively. In each group, 47 patients received bevacizumab as part of the systemic treatment.

After a median follow-up of 69.8 months, overall survival was significantly higher in the group of patients who underwent secondary CRS, with a median overall survival of 53.7 months versus 46.0 months (HR 0.75, 95% CI 0.59-0.96, p=0.02). Median progression-free survival was also superior after secondary CRS (HR 0.66, 95% CI 0.54-0.82, 18.4 vs. 14.0 months). Subgroup analysis, considering age, initial disease stage, histological subtype, the administration of maintenance therapy and duration of platinum-free interval, did not identify patients who did not benefit from secondary CRS. Complete CRS when compared with incomplete CRS was associated with a highly increased median overall survival (61.9 months, 95% CI 55.3-78.9 vs. 27.7 months, 95% CI 23.5-38.7). Notably, the median overall survival in non-operated patients was significantly higher (46.0 months, 95% CI 39.5-52.6) than the patients with incompletely resected recurrent disease. The median progression-free survival was almost two times higher after complete versus after incomplete CRS, with non-operated patients exhibiting a slightly higher progression-free survival than the patients in whom complete CRS could not be achieved. Regarding quality-of-life analysis, there were no substantial differences at 6 and 12 months after randomization. In the group of patients who underwent secondary CRS, the insomnia and constipation score were slightly higher at 6 months, but similar at 12 months. This might be attributed to the fact that at 6 months more patients in the CRS group were still receiving chemotherapy (38% vs. 11%).

### Comparison of randomized studies

In two of the three randomized trials (31, 32), the addition of secondary CRS to systemic chemotherapy appeared to be beneficial in patients with platinum-sensitive recurrence of ovarian cancer. In all three studies (30–32), secondary CRS was associated with acceptable surgical morbidity and did not appear to have a negative impact on quality of life. The median follow-up period was much longer in the DESKTOP III trial (69.8 months) than the GOG-0213 trial (48.1 months) and SOC-1 trial (36.0 months), making its results possibly more consistent. Progression-free survival was significantly improved by secondary CRS in the SOC-1 and DESKTOP III trials (31, 32), while in the GOG-0213 trial (30) no significant impact, neither negative nor positive, was observed after secondary CRS. Overall survival was significantly improved in the DESKTOP III (32), while in the SOC-1 trial (31) secondary CRS had no effect on overall survival, but the data were considered still immature for definite overall survival analysis and the high cross-over rate from the no surgery group to surgery at subsequent relapses might extend the median overall survival in the no surgery group and consequently result in limited statistical power to demonstrate potentially a reduced overall survival for the non-surgery group. In the GOG-0213 trial (30), no overall survival benefit was observed for secondary CRS. The discrepancy between GOG-0213 study (30) and the DESKTOP III trial (32) regarding the 3-year overall survival, with the GOG-0213 study exhibiting a lower rate in the complete secondary CRS group (76% vs. 84%) and at the same time a much higher in the no CRS arm (75% vs. 62%), suggests that there were some fundamental differences in the patient and treatment profile across the studies.

The lack of improvement of overall and progression-free survival in the GOG-0213 trial (30) may call into question the merit of secondary CRS in patients with platinum-sensitive ovarian cancer recurrence that appears preoperatively to be completely resectable. However, as discussed by the investigators, various factors may have diluted and masked an incremental benefit from secondary CRS. Firstly, the patients enrolled had considerably limited tumor load, with more than half of the patients having only one or two sites involved. In the GOG-0213 study (30) only 5% of the patients had peritoneal carcinomatosis, whereas in the SOC-1 (31) and the DESKTOP III trials (32) two third of the patients presented with multifocal disease relapse, including peritoneal carcinomatosis. The overall survival after secondary CRS is considerable higher when a single site is involved when compared with the case of multiple lesions or carcinomatosis. In a series of the Memorial Sloan Kettering Cancer Centre (22), secondary CRS for a single-site lesion multiple lesions and carcinomatosis (≥20 nodules) resulted in a median overall survival of 60, 42 and 28 months, respectively. Secondly, the patients in the GOG-0213 study had substantially platinum-sensitive disease, with a median platinum-free interval of 20.4 months, which is expected to make systemic treatment more effective. Thirdly, in the GOG-0213 trial (30) 84% of the patients received also bevacizumab, whereas in the SOC-1 (31) and the DESKTOP III trials (32) only in 1% and 23% of the patients this biological agent was administered. The highly effective systemic treatment regimen leading to a median overall survival of the entire study population being almost three times longer than expected, may definitely have diluted an independent effect of secondary CRS. Among the small group of patients who did not initially receive bevacizumab, secondary CRS was associated with worse overall survival. However, it is unknown who of the patients received the effective bevacizumab at a later point of treatment, resulting potentially in a treatment imbalance that could affect overall survival outcome. Whereas after secondary CRS the progression-free survival was slightly, non-significantly, better in the entire group and even statistically significantly better in the large subgroup of patients treated by paclitaxel-carboplatin and bevacizumab, overall survival was not improved by secondary CRS. Extended post-progression survival by improved clinical care and highly effective consecutive treatment regimens may have diluted the effect of secondary CRS measured according to progression-free survival by reducing statistical power to assess overall survival and enabling a higher probability of intervening treatment (35, 36). The differences in disease burden, use of biological agents and maintenance regimens across the three studies make a direct comparison very challenging.

Differences in outcome between the trials may also be attributed to the lack of standardization of surgical technique and surgical quality assurance among the participating centers as well as the difference in patient selection, causing heterogeneity of the study cohorts. In the GOG-0213 trial (30), the percentage of complete CRS was 67%, while in the other studies 77% (31) and 76% (32). The surgical skill and the ability of achieving complete CRS may differ considerably among centers and countries (37). In the GOG-0213 (30) participated 51 centers, from which 18 with 5 cases or less. Low volume centers may have more difficulty in achieving complete CRS (see ‘Referral centers’). The substantial difference in selection of patient for potentially complete secondary CRS among the randomized trials will be discussed below.

## Patient selection and prediction models

Appropriate patient selection is of paramount importance, performing secondary CRS only in those patients who may benefit and omitting secondary CRS in those who are not considered to benefit, avoiding unnecessary risk of surgical morbidity and costs. Firstly, patients should have platinum-sensitive disease, i.e being diagnosed with recurrence at least 6 months after the last platinum-based primary chemotherapy. Secondary CRS is generally not offered for resistant disease with evidence of progression during first line platinum-based chemotherapy (platinum-refractory), or recurrent disease within less than six months of completion of primary treatment (platinum-resistant). These women typically have poor prognosis and do not benefit from further surgical attempts at CRS (38, 39). Even if it has been possible to perform optimal or complete CRS, contrary to the case of ‘platinum-sensitive’ recurrent disease surgical treatment cannot be completed with effective chemotherapy. Hence, these patients may be exposed to unnecessary surgical morbidity and impairment of quality of life, without any significant survival benefit and are not to be considered candidates for secondary CRS.

Secondly, it appeared from above mentioned randomized trials (30–32) that only patients in whom complete CRS was achieved may benefit. Complete CRS, when compared with incomplete CRS, was associated with improved overall survival in the SOC-1 (31) and DESKTOP III (32) trials and improved progression-free survival in all three randomized trials (30, 31, 32). In the SOC-1 (31) and DESKTOP III (32) trials, patients who had undergone incomplete CRS, when compared with those receiving systemic treatment alone, exhibited a similar or slightly worse progression-free survival and even a significantly worse overall survival. In the GOG-0213, such a comparative analysis was not reported. This reduced survival in patients with incomplete CRS most probably reflects the aggressive biological behavior of the recurrent disease that prohibited complete CRS.

In a recent meta-analysis (40), the impact of the quality of secondary CRS on the survival of patients with platinum-sensitive recurrent ovarian cancer was studied. The meta-analysis comprised of 36 studies, published between 1995 and 2021, and a total of 2,805 patients. The majority of studies included were of retrospective nature. The median major surgical complication rate was 16.4% (0-44%), whereas a mean 30-day postoperative mortality of 0.7% was recorded. A significant heterogeneity among the studies was observed. The definition of optimal CRS varied considerably, from residual disease smaller than 0.25 cm to even residual tumor up to 2.5 cm. The median rate of complete and optimal CRS was 69.8% (9.4-100%) and 85.7% (43.5-100%), respectively. A meta-regression analysis to determine the cause of heterogeneity demonstrated the proportion of complete and optimal CRS to be statistically significant. Nevertheless, complete and optimal CRS were independent significant moderators of overall survival (p<0.001 and p=0.04, respectively). Studies with a complete CRS rate of higher than 70% reported a pooled overall survival rate of 65% in comparison with 46% in studies with an optimal CRS rate higher than 70% or less. For a cut off rate of 85% optimal CRS, the pooled overall survival rates were 63% and 47%, respectively. In multivariable analysis, with adjustment of the other variables, an increase of 10% in complete and optimal CRS was associated with respectively an increase of 8.97% and 7.04% in median overall survival. Hence, when secondary CRS is performed, a maximal effort should be made to accomplish complete or optimal disease resection in order to improve survival in patients with platinum-sensitive ovarian cancer recurrence. During the progress in systemic treatment the benefit of secondary CRS appeared to exist even more obviously in more recent years (p<0.001). For each 1-year increase in year of publication of the study, overall survival increased independently with 3.11% and 3.49% after complete and optimal CRS, respectively.

From the above it appeared of paramount importance to identify preoperatively the patients in whom complete or optimal secondary CRS can be performed, offering those patients the probable benefit of secondary CRS and avoiding potential surgical morbidity and costs in those who may not benefit since complete or optimal secondary CRS seems unfeasible. Various models for the prediction of complete secondary CRS in patients with recurrent ovarian cancer have been developed in order to have an objective tool that is more effective than just the individual surgeon’s opinion (41). In the three randomized trials the criteria for patient selection with respect to the probability of complete secondary CRS differed. In the GOG-0213 trial (30), while patients with preoperative evidence of ascites and/or diffuse peritoneal carcinomatosis were excluded, the platinum-sensitive recurrent disease was just ‘deemed by the investigator to amenable to complete gross resection’. With a considerably limited initial tumor load, as earlier mentioned, a complete CRS was reported in 67% of the cases. In the SOC-1 trial (31), completeness of CRS was predicted by a Tian or iMODEL score of ≤4.7 and, when the score was >4.7 and the tumor marker CA-125 >105 U/mL, by PET-CT imaging. As mentioned above, the Tian Score System, uses six variables, including FIGO stage, residual disease after primary surgery, platinum-free interval, ECOG performance status, serum level of CA-125 and presence of ascites at recurrence (33) (**Table 2**). In the original study (33), a value of ≤4.7 predicted a potentially complete resection rate of 53% vs. 20% for a higher score. In the SOC-1 study, complete secondary CRS was achieved in 77% of the cases, more frequently than in the GOG-0213 study. A recent retrospective, propensity score-matched analysis demonstrated that in Tian-model low-risk patients secondary CRS was associated with increased survival outcome when compared with chemotherapy only (42). In the DESKTOP III trial (32), the AGO (Arbeitsgemeinschaft Gynaekologische Onkologie) score had been used to select patients for secondary CRS and in 76% of the selected patients macroscopically complete resection of recurrent disease could be performed. This score was initially developed by the Descriptive Evaluation of preoperative Selection KriTeria for OPerability in recurrent OVARian cancer (DESKTOP OVAR) study (34). Retrospective analysis in databases from multiple centers determined objective selection criteria to identify patients with platinum-sensitive recurrent ovarian cancer that may benefit from secondary surgery. Complete secondary CRS was associated with a significantly longer median survival than incomplete secondary CRS (45.2 vs. 19.7 months, HR 3.7, 95% CI 2.27-6.05, p<0.0001). Variable associated with complete secondary CRS included ECOG performance status (0 vs. >0, p<0.001), FIGO stage at initial diagnosis (I/II vs. III/IV, p=0.036), residual tumor after primary CRS (absent vs. present, p<0.0001) and absence of ascites >500 ml (p<0.001). A positive AGO score, being a combination of performance status ECOG 0, complete primary CRS in the past (or when data not available initial FIGO I/II disease), and absence of ascites >500 ml, could predict complete secondary CRS in 79% of the patients with platinum-sensitive ovarian cancer relapse (**Table 3**). In the DESKTOP II trial (43), this AGO score was prospectively validated to predict completeness of secondary CRS. Two-hundred and sixty-one of the 516 screened patients (51%) had a positive AGO score. Complete secondary CRS was achieved in 76% of the 129 patients with a positive AGO score who underwent secondary surgery, while surgical morbidity and mortality were acceptable. Consequently, this prospective study verified the value of the AGO score in patient selection for secondary CRS. However, in an exploratory analysis by the same group the complete CRS rate for a positive AGO score was 89.3% and for a negative AGO score still 66.7%, underlining its suboptimal negative predictive value (44). Another prediction model for complete secondary CRS that has been externally validated in clinical studies has been developed at the Memorial Sloan Kettering Cancer Centre (22). The MSK Criteria are based on disease-free interval (6-12, 12-30, >30 months), single vs. multiple recurrence sites and evidence of carcinomatosis (≥20 nodules) (**Table 4**). The effectiveness of those three prediction models have been tested retrospectively and compared with each other in various studies (45–49). In patients with platinum-sensitive recurrent ovarian cancer who were initially treated with primary systemic chemotherapy and interval CRS instead of primary CRS followed by systemic chemotherapy, these predictive models have similar efficacy (50). While their positive predictive value for complete CRS was generally high (73-86%), unfortunately the false negative rate of those models was relatively high (55-70%). Hence, these prediction models may be too strict and exclude patients who may have a chance of successful secondary CRS. Consequently, further studies are warranted so as not to prohibit patients from undergoing potential life-extending surgery. The addition of preoperative imaging and/or staging laparoscopy to the criteria of those prediction models may be beneficial.

**Table 4:** The MSK criteria (22).

Disease-free interval	Single site of recurrence	Multiple sites of recurrence but no carcinomatosis	Peritoneal carcinomatosis
6-12 months	Offer sCRS	Consider sCRS	No sCRS
12-30 months	Offer sCRS	Offer sCRS	Consider sCRS
>30 months	Offer sCRS	Offer sCRS	Offer sCRS

MSK, Memorial Sloan Kettering; sCRS, secondary cytoreductive surgery, * ≥ tumor 20 tumor nodules at time of surgery.

Regarding the preoperative radiological workup, contrast enhanced computed tomography (CT) is usually the technique of choice for follow-up of patients with ovarian cancer, but its efficacy is limited by its low soft-tissue contrast in evaluating disease in the pelvis and on visceral surfaces (51). Magnetic resonance imaging (MRI) has excellent soft-tissue resolution and the capacity to discriminate between post-treatment changes and tumor recurrence, but its diagnostic accuracy is limited in small-volume recurrent lesions and in sites where the lesions are contiguous to tissues with similar signal intensity (52). Diffuse weighted imaging MRI seems promising to identify small peritoneal and nodal lesions (53). Combining anatomical and functional imaging through positron emission imaging (PET)/CT may help evaluate patients with suspected ovarian cancer recurrence but negative or indeterminate CT findings. In a recent meta-analysis of 34 studies (54), the pooled area under the curve (AUC) of PET/CT for detecting ROC was significantly higher than that of CT or MRI. PET-CT and staging laparoscopy may be helpful in identification of patients in which complete CRS may be feasible (55–57). Staging laparoscopy is feasible in the vast majority of patients with recurrent ovarian cancer, despite the major abdominal surgery that usually has preceded (55). While their negative and positive predictive value, sensitivity and specificity in assessing the possibility of complete CRS are quite similar, PET-CT and staging laparoscopy should be considered complementary modalities (56). The combination of these preoperative examinations seems better than the AGO-score in patient selection for complete or optimal CRS. In a comparative study (55), approximately 20% of patients with negative AGO score achieved actually successful secondary CRS after preoperative evaluation with PET–CT and staging laparoscopy, whereas almost one of three positive AGO score patients, who had however a negative assessment with PET-CT and staging laparoscopy, would be submitted to an unnecessary explorative laparotomy.

Moreover, the identification and incorporation of predictive biomarkers to tailor the medical and surgical approach, including secondary CRS, is paramount to the success of treatment of recurrent ovarian cancer. BRCA mutation status is a potential selection parameter for secondary CRS in the future, although its role is still to be defined. Women with BRCA mutation are likely to receive a new emerging treatment with PARP inhibitors that has notably improved progression-free survival, as mentioned previously. In a recent multicenter study (58), germline BRCA mutation carriers were more likely to undergo secondary cytoreduction. This may be mediated in part by lower volume disease at recurrence. In a multicenter study (59) to assess the role of BRCA mutation status in personalizing the management of recurrent ovarian cancer, BRCA mutation patients had the best prognosis regardless of secondary CRS, whereas post-recurrence survival in BRCA wild type women was improved by complete secondary CRS. In another study, however, the benefit of secondary CRS was similar for both groups of patients (60). Similarly, in a similar case-control study (61) ovarian cancer patients with a BRCA mutation who underwent secondary CRS and subsequently received chemotherapy and a PARP inhibitor experienced a better survival than those who received chemotherapy and a PARP inhibitor only. Moreover, resection of hepatic recurrences, isolated or with concomitant peritoneal disease, seem to be associated with a favorable outcome only in patients with BRCA mutations (62). As mentioned previously, salvage lymphadenectomy as secondary CRS seems beneficial independently of BRCA mutational status (25).

## Referral centers

CRS is a demanding and complex procedure, which may include specific surgical techniques such as peritonectomies, may require a multidisciplinary surgical team and may expose the patient to an increased risk of surgical morbidity. The procedure is associated with a long learning curve for a center in order to achieve a high complete CRS rate with synchronously low major surgical morbidity and mortality, less blood loss, shorter operation time and shorter hospital stay (63). The number of cases to overcome the learning curve varied from 130 to 220 in series of patients with peritoneal carcinomatosis from various origins, of whom most underwent besides CRS also HIPEC (63–67). In another study (68), the learning curve with respect to operation time and total blood loss was considered significantly longer for high-complexity procedures with bowel resection and upper abdominal surgery for primary advanced ovarian cancer than for moderate-complexity procedures. While the learning curve for the complete primary CRS rate was not examined, no typical learning curve was observed concerning the occurrence of severe complications. A mentorship model by surgeons with a large experience and knowledge of CRS should be paramount to reduce the prolonged learning curve for the achievement of proficiency considering radicality and safety (63, 69–71).

Advanced surgical skills as applied in referral centers might be one step towards increasing the complete CRS rate and consequently the proportion of patients who might benefit from surgery for primary and recurrent ovarian cancer. In primary surgery for advanced ovarian cancer, a paradigm shift toward more aggressive surgery as well as training in and incorporation of extensive upper abdominal procedures resulted in a higher chance on complete CRS in referral centers (71–74).

There are, as far as we know, no data published regarding learning curves and surgical skills in secondary CRS for recurrent ovarian cancer. While expertise and surgical skills are important in order to offer the highest chance of complete CRS and synchronously low surgical morbidity in primary ovarian cancer, this should be even more the case for secondary CRS in women who have already been operated, usually extensively, for primary ovarian cancer. The maximal effort to achieve complete secondary CRS may require collaboration of various surgical specialists such gynecological and surgical oncologists, gastrointestinal surgeons, urologists, hepatobiliary surgeons, vascular surgeons and other. Such a multidisciplinary surgical team is preferably created in a referral center in order to obtain adequate experience.

## Secondary cytoreductive surgery and HIPEC

CRS is also mandatorily performed when intraperitoneal chemotherapy is applied for ovarian cancer. Intraperitoneal chemotherapy has a pharmacological advantage above systemic, intravenous chemotherapy (75–77). Due to the slow absorption of chemotherapeutic drugs from the peritoneal cavity and the first-pass effect in the liver, a high intraperitoneal drug concentration can be achieved with simultaneously low systemic drug toxicity. Intraoperative application of HIPEC assures optimal exposure of the drug to the entire seroperitoneal surface and early treatment of (microscopic) residual disease before re-growth can occur. Heating the drug solution as in intraoperative hyperthermic intraperitoneal chemotherapy (HIPEC) increases the efficacy of intraperitoneally administered drugs, while heat itself may have a direct cytotoxic effect. However, the penetration depth of intraperitoneally delivered drugs into tumor nodules is very limited and hence thorough resection of macroscopic peritoneal disease, i.e. complete or optimal CRS, should precede intraperitoneal chemotherapy (75, 76).

During the last decades, CRS and HIPEC has been applied in various primary and secondary peritoneal malignancies, among which advanced ovarian cancer (78, 79). Only a few randomized trials on HIPEC for ovarian cancer have been reported. The recently published Korean randomized KOV-HIPEC-1 trial (80) did not show benefit of the addition of HIPEC to primary CRS and systemic chemotherapy for primary advanced ovarian cancer. In the Dutch multicenter randomized OVHIPEC trial (81), in the Spanish multicenter randomized CARCINOHIPEC trial (82) and in the subgroup analysis of the KOV-HIPEC-1 trial (80), an evident benefit was observed for the addition of HIPEC to interval CRS after primary chemotherapy in primary ovarian cancer. In the largest of the randomized trials, the Dutch OVHIPEC study (81), only patients with at least stable disease during primary chemotherapy and complete or optimal interval CRS were enrolled in the study.

Regarding secondary CRS and HIPEC for relapsed ovarian cancer, a Greek single-center randomized trial (83) reported improved overall survival for the patients who underwent secondary CRS and HIPEC (n=60), both in platinum-sensitive and platinum-resistant disease, when compared with CRS only (n=60). All received systemic chemotherapy postoperatively. However, the validity of the study has been contested due to significant shortcomings: the randomization process was not described in detail, primary end points were not clearly defined, there was no information provided regarding disease-free survival, complications, postoperative systemic chemotherapy and follow-up, and the study had not been registered in an international clinical trial database (84). Moreover, others raised that the statistical analysis performed in the study was not clearly described and inappropriately applied, mean instead of median OS was used, reported data were inconsistent with provided graphics and their recalculation of the statistics demonstrated the outcome after HIPEC to be not statistically significantly superior to the control group (85, 86). Most recently, the results of the Memorial Sloan Kettering (MSK) Team Ovary randomized phase II study have been reported (87). Ninety-eight patients with ovarian cancer recurrence were randomly assigned to secondary CRS and HIPEC or secondary CRS only, in both groups followed by systemic chemotherapy. Although complete CRS had been more frequently achieved in the HIPEC group (94% vs. 82%), the addition of HIPEC to secondary CRS did not improve disease progression-free or overall survival. In both randomized trials secondary CRS was performed in both arms and therefore a potential partial role of secondary CRS cannot be determined in this setting.

## Conclusions and future directions

As discussed above, two of the three recently reported randomized trials (31, 32) have demonstrated that a definite role exists for secondary CRS in patients with recurrent ovarian cancer with respect to survival improvement, but only when complete resection of macroscopic disease can be achieved. Complete CRS was associated with significantly better survival outcome than after incomplete CRS in recent randomized trials and meta-analyses, with incomplete CRS be associated with worse survival than chemotherapy only (19, 30–32, 40). Patient selection is of paramount importance to identify those patients in whom complete secondary CRS seems to be feasible. Various models for this patient selection have been developed with an adequate preoperative prediction of achievement of complete secondary CRS (22, 33, 34, 41). However, the negative predictive rate is relatively high. Hence, these prediction models may be too strict and exclude patients who may have a chance of successful secondary CRS. Consequently, further studies are warranted to improve these prediction models with respect to their negative predictive value, so that patients who may benefit are not prohibited from undergoing potential life-extending surgery.

CRS is a demanding and complex procedure with a long learning curve to accomplish a high complete CRS rate and low surgical morbidity (63). When performed by experienced teams, secondary CRS is safe and without a negative impact on quality of life (40). Therefore, secondary CRS is preferably performed in referral centers with ample experience. It is crucial to develop standardized training programs and mentorships to shorten the long learning process to reduce morbidity and mortality, and improve oncologic outcomes (63, 69–71). The impact of the multidisciplinary effort in the treatment of ovarian cancer relapse is being indirectly reflected by the increasing survival outcomes in more recently published studies on secondary CRS (40), which is result of the significant improvement in both surgically and systemically management over the last decades.

The role of secondary CRS should be continuously reviewed considering the changing systemic treatment of patients with ovarian cancer recurrence over time. A well-designed biomarker-driven randomized trial with prespecified subgroup analysis seems rather ambitious, but will certainly reveal further the true effect of secondary CRS in the various ovarian cancer subgroups. As discussed previously, some recent retrospective studies have assessed the impact of biological features, such as the BRCA status and the use of PARP inhibitors, on the potential benefit of secondary CRS in patients with platinum-sensitive ovarian cancer relapse with yet inconclusive data (25, 58–62). The results of the randomized phase II SGOG SOC-3 study (88) on the benefit of CRS before receiving platinum-based chemotherapy and a PARP inhibitor in patients with a secondary platinum-sensitive ovarian recurrence are eagerly awaited. Further studies should be conducted to determine the benefits of secondary CRS with respect to the molecular characteristics (BRCA or homologous recombination deficiency status) and the use of PARP inhibitors and/or bevacizumab. The forthcoming research trend is to achieve a more accurate, individualized treatment approach of recurrent ovarian cancer.

## Author contributions

All of the coauthors listed meet the criteria for authorship. EB provided the concept and guidance. EB and EA reviewed the literature. EB wrote and DM critically reviewed the manuscript. All authors contributed to the article and approved the submitted version.

## Funding

The company Anastasios Mavrogenis S.A., Trade of Medical Medicines, was so kind to pay the article processing fees.

## Conflict of interest

This study received funding from Anastasios Mavrogenis S.A. The funder was not involved in the study design, collection, analysis, interpretation of data, the writing of this article or the decision to submit it for publication.

The authors declare that the research was conducted in the absence of any commercial or financial relationships that could be construed as a potential conflict of interest.

## Publisher’s note

All claims expressed in this article are solely those of the authors and do not necessarily represent those of their affiliated organizations, or those of the publisher, the editors and the reviewers. Any product that may be evaluated in this article, or claim that may be made by its manufacturer, is not guaranteed or endorsed by the publisher.

## References

[1] Sung H Ferlay J Siegel RL Laversanne M Soerjomataram I Jemal A. Global cancer statistics 2020: GLOBOCAN estimates of incidence and mortality worldwide for 36 cancers in 185 countries. CA Cancer J Clin (2021) 71(3):209–49. doi: 10.3322/caac.21660 33538338

[2] Siegel RL Miller KD Fuchs HE Jemal A. Cancer statistics, 2022. CA Cancer J Clin (2022) 72(1):7–33. doi: 10.3322/caac.21708 35020204

[3] du Bois A Quinn M Thigpen T Vermorken J Avall-Lundqvist E Bookman M. 2004 Consensus statements on the management of ovarian cancer: Final document of the 3rd international gynecologic cancer intergroup ovarian cancer consensus conference (GCIG OCCC 2004). Ann Oncol (2005) 16 Suppl 8:viii7–12. doi: 10.1093/annonc/mdi961 16239238

[4] Querleu D Planchamp F Chiva L Fotopoulou C Barton D Cibula D. European Society of gynaecological oncology (ESGO) guidelines for ovarian cancer surgery. Int J Gynecol Cancer (2017) 27(7):1534–42. doi: 10.1097/IGC.0000000000001041 30814245

[5] Burger RA Brady MF Bookman MA Fleming GF Monk BJ Huang H. Incorporation of bevacizumab in the primary treatment of ovarian cancer. N Engl J Med (2011) 365(26):2473–83. doi: 10.1056/NEJMoa1104390 22204724

[6] Perren TJ Swart AM Pfisterer J Ledermann JA Pujada-Lauraine E Kristensen G. A phase 3 trial of bevacizumab in ovarian cancer. N Engl J Med (2011) 365(26):2484–96. doi: 10.1056/NEJMoa1103799 22204725

[7] Banerjee S Moore KN Colombo N Scambia G Kim BG Oaknin A. Maintenance olaparib for patients with newly diagnosed advanced ovarian cancer and a BRCA mutation (SOLO1/GOG 3004): 5-year follow-up of a randomised, double-blind, placebo-controlled, phase 3 trial. Lancet Oncol (2021) 22(12):1721–31. doi: 10.1016/S1470-2045(21)00531-3 34715071

[8] González-Martín A Pothuri B Vergote I Depont-Christensen R Graybill W Mirza MR. Niraparib in patients with newly diagnosed advanced ovarian cancer. N Engl J Med (2019) 381(25):2391–402. doi: 10.1056/NEJMoa1910962 31562799

[9] Coleman RL Fleming GF Brady MF Swisher EM Steffensen KD Friedlander M. Veliparib with first-line chemotherapy and as maintenance therapy in ovarian cancer. N Engl J Med (2019) 381(25):2403–15. doi: 10.1056/NEJMoa1909707 PMC6941439 31562800

[10] Ray-Coquard I Pautier P Pignata S Pérol D González-Martín A Berger R. Olaparib plus bevacizumab as first-line maintenance in ovarian cancer. N Engl J Med (2019) 381(25):2416–28. doi: 10.1056/NEJMoa1911361 31851799

[11] Lorusso D Marchetti C Conte C Giudice E Bolomini G Vertechy L. Bevacizumab as maintenance treatment in BRCA mutated patients with advanced ovarian cancer: A large, retrospective, multicenter case-control study. Gynecol Oncol (2020) 159(1):95–100. doi: 10.1016/j.ygyno.2020.07.022 32703631

[12] Bristow RE Tomacruz RS Armstrong DK Trimble EL Montz FJ. Survival effect of maximal cytoreductive surgery for advanced ovarian carcinoma during the platinum era: a meta-analysis. J Clin Oncol (2002) 20(5):1248–59. doi: 10.1200/JCO.2002.20.5.1248 11870167

[13] Chang SJ Hodeib M Chang J Bristow RE. Survival impact of complete cytoreduction to no gross residual disease for advanced-stage ovarian cancer: A meta-analysis. Gynecol Oncol (2013) 130(3):493–8. doi: 10.1016/j.ygyno.2013.05.040 23747291

[14] Goldie JH Coldman AJ. A mathematic model for relating the drug sensitivity of tumors to their spontaneous mutation rate. Cancer Treat Rep (1979) 63:1727–33. 526911

[15] Norton L Simon R. Tumor size, sensitivity to therapy, and design of treatment schedules. Cancer Treat Rep (1977) 61:1307–17. 589597

[16] Sundar S Wu J Hillaby K Yap J Lilford R. A systematic review evaluating the relationship between progression free survival and post progression survival in advanced ovarian cancer. Gynecol Oncol (2012) 125(2):493–9. doi: 10.1016/j.ygyno.2011.12.420 22155676

[17] Luvero D Plotti F Aloisia A Montera R Terranova C de Cicco Nardone C. Ovarian cancer relapse: From the latest scientific evidence to the best practice. Crit Rev Oncol Hematol (2019) 140:28–38. doi: 10.1016/j.critrevonc.2019.05.014 31176270

[18] Gallotta V Jeong SY Conte C Trozzi R Cappuccio S Moroni R. Minimally invasive surgical staging for early stage ovarian cancer: A long-term follow up. Eur J Surg Oncol (2021) 47(7):1698–704. doi: 10.1016/j.ejso.2021.01.033 33573854

[19] Bristow RE Puri I Chi DS. Cytoreductive surgery for recurrent ovarian cancer: A meta-analysis. Gynecol Oncol (2009) 112:265–74. doi: 10.1016/j.ygyno.2008.08.033 18937969

[20] Bommert M Harter P Heitz F du Bois A. When should surgery be used for recurrent ovarian carcinoma? Clin Oncol (R Coll Radiol) (2018) 30:493–7. doi: 10.1016/j.clon.2018.04.006 29743148

[21] Zang RY Harter P Chi DS Sehouli J Jiang R Tropé CG. Predictors of survival in patients with recurrent ovarian cancer undergoing secondary cytoreductive surgery based on the pooled analysis of an international collaborative cohort. Br J Cancer (2011) 105(7):890–6. doi: 10.1038/bjc.2011.328 PMC3185944 21878937

[22] Chi DS McCaughty K Diaz JP Huh J Schwabenbauer S Hummer AJ. Guidelines and selection criteria for secondary cytoreductive surgery in patients with recurrent, platinum-sensitive epithelial ovarian carcinoma. Cancer (2006) 106(9):1933–9. doi: 10.1002/cncr.21845 16572412

[23] Al Rawahi T Lopes AD Bristow RE Bryant A Elattar A Chattopadhyay S. Surgical cytoreduction for recurrent epithelial ovarian cancer. Cochrane Database Syst Rev (2013) 2013(2):CD008765. doi: 10.1002/14651858.CD008765.pub3 PMC6457850 23450588

[24] Gallotta V Conte C Giudice MT Nero C Vizzielli G Gueli Alletti S. Secondary laparoscopic cytoreduction in recurrent ovarian cancer: A large, single-institution experience. J Minim Invasive Gynecol (2018) 25(4):644–50. doi: 10.1016/j.jmig.2017.10.024 29081384

[25] Gallotta V Bruno M Conte C Giudice MT Davià F Moro F. Salvage lymphadenectomy in recurrent ovarian cancer patients: Analysis of clinical outcome and BRCA1/2 gene mutational status. Eur J Surg Oncol (2020) 46(7):1327–33. doi: 10.1016/j.ejso.2020.01.035 32085925

[26] Loverro M Ergasti R Conte C Gallitelli V Nachira D Scaglione G. Minimally invasive secondary cytoreductive surgery for superficial celiac and cardio-phrenic isolated nodal recurrence of ovarian cancer. Ann Surg Oncol (2022) 29(4):2603–4. doi: 10.1245/s10434-021-11267-5 35000090

[27] Gallotta V Giudice MT Conte C Sarandeses AV D'Indinosante M Federico A. Minimally invasive salvage lymphadenectomy in gynecological cancer patients: A single institution series. Eur J Surg Oncol (2018) 44(10):1568–72. doi: 10.1016/j.ejso.2018.08.006 30170883

[28] Tinelli G Cappuccio S Parente E Fagotti A Gallotta V Conte C. Resectability and vascular management of retroperitoneal gynecological malignancies: A large single-institution case-series. Anticancer Res (2017) 37(12):6899–906. doi: 10.21873/anticanres.12153 29187471

[29] Baert T Ferrero A Sehouli J O'Donnell DM González-Martín A Joly F. The systemic treatment of recurrent ovarian cancer revisited. Ann Oncol (2021) 32(6):710–25. doi: 10.1016/j.annonc.2021.02.015 33675937

[30] Coleman RL Spirtos NM Enserro D Herzog TJ Sabbatini P Armstrong DK. Secondary surgical cytoreduction for recurrent ovarian cancer. N Engl J Med (2019) 381(20):1929–39. doi: 10.1056/NEJMoa1902626 PMC6941470 31722153

[31] Shi T Zhu J Feng Y Tu D Zhang Y Zhang P. Secondary cytoreduction followed by chemotherapy versus chemotherapy alone in platinum-sensitive relapsed ovarian cancer (SOC-1): A multicentre, open-label, randomised, phase 3 trial. Lancet Oncol (2021) 22(4):439–49. doi: 10.1016/S1470-2045(21)00006-1 33705695

[32] Harter P Sehouli J Vergote I Ferron G Reuss A Meier W. Randomized trial of cytoreductive surgery for relapsed ovarian cancer. N Engl J Med (2021) 385(23):2123–31. doi: 10.1056/NEJMoa2103294 34874631

[33] Tian WJ Chi DS Sehouli J Tropé CG Jiang R Ayhan A. A risk model for secondary cytoreductive surgery in recurrent ovarian cancer: an evidence-based proposal for patient selection. Ann Surg Oncol (2012) 19(2):597–604. doi: 10.1245/s10434-011-1873-2 21732142

[34] Harter P du Bois A Hahmann M Hasenburg A Burges A Loibl S. Surgery in recurrent ovarian cancer: the arbeitsgemeinschaft gynaekologische onkologie (AGO) DESKTOP OVAR trial. Ann Surg Oncol (2006) 13(12):1702–10. doi: 10.1245/s10434-006-9058-0 17009163

[35] Broglio KR Berry DA. Detecting an overall survival benefit that is derived from progression-free survival. J Natl Cancer Inst (2009) 101(23):1642–9. doi: 10.1093/jnci/djp369 PMC4137232 19903805

[36] Morita S Sakamaki K Yin G. Detecting overall survival benefit derived from survival postprogression rather than progression-free survival. J Natl Cancer Inst (2015) 107(8):djv133. doi: 10.1093/jnci/djv133 25956357 PMC4609552

[37] Vergote I Tropé CG Amant F Kristensen GB Ehlen T Johnson N. Neoadjuvant chemotherapy or primary surgery in stage IIIC or IV ovarian cancer. N Engl J Med (2010) 363(10):943–53. doi: 10.1056/NEJMoa0908806 20818904

[38] Eisenkop SM Friedman RL Spirtos NM. The role of secondary cytoreductive surgery in the treatment of patients with recurrent epithelial ovarian carcinoma. Cancer (2000) 88(1):144–53. doi: 10.1002/(sici)1097-0142(20000101)88:1<144::aid-cncr20>3.3.co;2-o 10618617

[39] Tebes SJ Sayer RA Palmer JM Tebes CC Martino MA Hoffman MS. Cytoreductive surgery for patients with recurrent epithelial ovarian carcinoma. Gynecol Oncol (2007) 106(3):482–7. doi: 10.1016/j.ygyno.2007.04.006 17590420

[40] Baek MH Park EY Ha HI Park SY Lim MC Fotopoulou C. Secondary cytoreductive surgery in platinum-sensitive recurrent ovarian cancer: A meta-analysis. J Clin Oncol (2022) 20;40(15):1659–70. doi: 10.1200/JCO.21.02085 35188810

[41] Jiang C Li Z. Prediction models for complete resection in secondary cytoreductive surgery of patients with recurrent ovarian cancer. Front Oncol (2021) 11:674637. doi: 10.3389/fonc.2021.674637 34631517 PMC8496933

[42] So M Miyamoto T Murakami R Abiko K Hamanishi J Baba T. The efficacy of secondary cytoreductive surgery for recurrent ovarian, tubal, or peritoneal cancer in tian-model low-risk patients. J Gynecol Oncol (2019) 30(6):e100. doi: 10.3802/jgo.2019.30.e100 31576692 PMC6779625

[43] Harter P Sehouli J Reuss A Hasenburg A Scambia G Cibula D. Prospective validation study of a predictive score for operability of recurrent ovarian cancer: The multicenter intergroup study DESKTOP II. a project of the AGO kommission OVAR, AGO study group, NOGGO, AGO-Austria, and MITO. Int J Gynecol Cancer (2011) 21(2):289–95. doi: 10.1097/IGC.0b013e31820aaafd 21270612

[44] Harter P Beutel B Alesina PF Lorenz D Boergers A Heitz F. Prognostic and predictive value of the arbeitsgemeinschaft gynaekologische onkologie (AGO) score in surgery for recurrent ovarian cancer. Gynecol Oncol (2014) 132(3):537–41. doi: 10.1016/j.ygyno.2014.01.027 24462732

[45] Cowan RA Eriksson A Jaber SM Zhou Q Iasonos A Zivanovic O. A comparative analysis of prediction models for complete gross resection in secondary cytoreductive surgery for ovarian cancer. Gynecol Oncol (2017) 145(2):230–5. doi: 10.1016/j.ygyno.2017.02.010 28285846

[46] Bogani G Tagliabue E Signorelli M Ditto A Martinelli F Chiappa V. A score system for complete cytoreduction in selected recurrent ovarian cancer patients undergoing secondary cytoreductive surgery: predictors and nomogram-based analyses. J Gynecol Oncol (2018) 29(3):e40. doi: 10.3802/jgo.2018.29.e40 29533023 PMC5920224

[47] van de Laar R Massuger LF Van Gorp T IntHout J Zusterzeel PL Kruitwagen RF. External validation of two prediction models of complete secondary cytoreductive surgery in patients with recurrent epithelial ovarian cancer. Gynecol Oncol (2015) 137(2):210–5. doi: 10.1016/j.ygyno.2015.02.004 25677063

[48] Janco JMT Kumar A Weaver AL McGree ME Cliby WA. Performance of AGO score for secondary cytoreduction in a high-volume U.S. center. Gynecol Oncol (2016) 141(1):140–7. doi: 10.1016/j.ygyno.2016.01.027 26836496

[49] Laga T Lambrechts S Laenen A Van Nieuwenhuysen E Han SN Vergote I. Positive DESKTOP and tian scores systems are adequate to predict optimal (r0) secondary debulking surgery in ovarian cancer, but a negative score does not preclude secondary surgery. Int J Gynecol Cancer (2018) 28(4):721–8. doi: 10.1097/IGC.0000000000001219 29561300

[50] Bizzarri N Marchetti C Conte C Loverro M Giudice MT Quagliozzi L. The impact of secondary cytoreductive surgery in platinum sensitive recurrent ovarian cancer treated with upfront neoadjuvant chemotherapy and interval debulking surgery. Gynecol Oncol (2022) 165(3):453–8. doi: 10.1016/j.ygyno.2022.03.024 35397918

[51] Nasser S Lazaridis A Evangelou M Jones B Nixon K Kyrgiou M. Correlation of pre-operative CT findings with surgical & histological tumor dissemination patterns at cytoreduction for primary advanced and relapsed epithelial ovarian cancer: A retrospective evaluation. Gynecol Oncol (2016) 143(2):264–69. doi: 10.1016/j.ygyno.2016.08.322 27586894

[52] Sanli Y Turkmen C Bakir B Iyibozkurt C Ozel S Has D. Diagnostic value of PET/CT is similar to that of conventional MRI and even better for detecting small peritoneal implants in patients with recurrent ovarian cancer. Nucl Med Commun (2012) 33:509–15. doi: 10.1097/MNM.0b013e32834fc5bf 22357440

[53] Hameeduddin A Sahdev A. Diffusion-weighted imaging and dynamic contrast-enhanced MRI in assessing response and recurrent disease in gynaecological malignancies. Cancer Imaging (2015) 15(1):3. doi: 10.1186/s40644-015-0037-1 25889065 PMC4432943

[54] Satoh Y Ichikawa T Motosugi U Kimura K Sou H Sano K. Diagnosis of peritoneal dissemination: comparison of 18F-FDG PET/CT, diffusion-weighted MRI, and contrast-enhanced MDCT. AJR Am J Roentgenol (2011) 196(2):447–53. doi: 10.2214/AJR.10.4687 21257899

[55] Fanfani F Monterossi G Fagotti A Gallotta V Costantini B Vizzielli G. Positron emission tomography-laparoscopy based method in the prediction of complete cytoreduction in platinum-sensitive recurrent ovarian cancer. Ann Surg Oncol (2015) 22(2):649–54. doi: 10.1245/s10434-014-4011-0 25155399

[56] Fagotti A Fanfani F Rossitto C Lorusso D De Gaetano AM Giordano A. A treatment selection protocol for recurrent ovarian cancer patients: the role of FDG-PET/CT and staging laparoscopy. Oncology (2008) 75(3-4):152–8. doi: 10.1159/000159266 18827492

[57] Conte C Fagotti A Avesani G Trombadori C Federico A D'Indinosante M. Update on the secondary cytoreduction in platinum-sensitive recurrent ovarian cancer: a narrative review. Ann Transl Med (2021) 9(6):510. doi: 10.21037/atm-20-4690 33850907 PMC8039681

[58] Yfat K Mariam K Mario B Hal H Dana J Lina S. Germline BRCA mutation carriers are more likely to undergo cytoreductive surgery for relapsed, platinum sensitive, ovarian cancer. Gynecol Oncol (2022) 22)00574-1:S0090–8258. doi: 10.1016/j.ygyno.2022.08.020 36154762

[59] Marchetti C De Leo R Musella A D'Indinosante M Capoluongo E Minucci A. BRCA mutation status to personalize management of recurrent ovarian cancer: A multicenter study. Ann Surg Oncol (2018) 25(12):3701–8. doi: 10.1245/s10434-018-6700-6 30128899

[60] Estati FL Pirolli R de Alencar VTL Ribeiro ARG Formiga MN Torrezan GT. Impact of BRCA1/2 mutations on the efficacy of secondary cytoreductive surgery. Ann Surg Oncol (2021) 28(7):3637–45. doi: 10.1245/s10434-020-09366-w 33221980

[61] Marchetti C Rosati A Scaletta G Pietragalla A Arcieri M Ergasti R. Secondary cytoreductive surgery in platinum-sensitive recurrent ovarian cancer before olaparib maintenance: Still getting any benefit? a case-control study. Gynecol Oncol (2019) 155(3):400–5. doi: 10.1016/j.ygyno.2019.09.020 31606285

[62] Gallotta V Conte C D'Indinosante M Capoluongo E Minucci A De Rose AM. Prognostic factors value of germline and somatic brca in patients undergoing surgery for recurrent ovarian cancer with liver metastases. Eur J Surg Oncol (2019) 45(11):2096–102. doi: 10.1016/j.ejso.2019.06.023 31227342

[63] Santullo F Abatini C Attalla El Halabieh M Ferracci F Lodoli C Barberis L. The road to technical proficiency in cytoreductive surgery for peritoneal carcinomatosis: Risk-adjusted cumulative summation analysis. Front Surg (2022) 9:877970. doi: 10.3389/fsurg.2022.877970 35662826 PMC9157764

[64] Smeenk RM Verwaal VJ Zoetmulder FAN. Learning curve of combined modality treatment in peritoneal surface disease. Br J Surg (2007) 94(11):1408–14. doi: 10.1002/bjs.5863 17631678

[65] Kusamura S Baratti D Deraco M. Multidimensional analysis of the learning curve for cytoreductive surgery and hyperthermic intraperitoneal chemotherapy in peritoneal surface malignancies. Ann Surg (2012) 255:348–56. doi: 10.1097/SLA.0b013e3182436c28 22202584

[66] Andréasson H Lorant T Påhlman L Graf W Mahteme H. Cytoreductive surgery plus perioperative intraperitoneal chemotherapy in pseudomyxoma peritonei: aspects of the learning curve. Eur J Surg Oncol (2014) 40:930–6. doi: 10.1016/j.ejso.2014.03.001 24656455

[67] Polanco PM Ding Y Knox JM Ramalingam L Jones H Hogg ME. Institutional learning curve of cytoreductive surgery and hyperthermic intraperitoneal chemoperfusion for peritoneal malignancies. Ann Surg Oncol (2015) 22:1673–9. doi: 10.1245/s10434-014-4111-x 25377640

[68] Nishikimi K Tate S Matsuoka A Shozu M. Learning curve of high-complexity surgery for advanced ovarian cancer. Gynecol Oncol (2020) 156(1):54–61. doi: 10.1016/j.ygyno.2019.10.034 31735352

[69] Chang KH Kazanowski M Staunton O Cahill RA Moran BJ Shields C. Mentored experience of establishing a national peritoneal malignancy programme - experience of first 50 operative cases. Eur J Surg Oncol (2017) 43(2):395–400. doi: 10.1016/j.ejso.2016.10.007 27955836

[70] Kusamura S González-Moreno S Nizri E Baratti D Guadagni S Guaglio M. Learning curve, training program, and monitorization of surgical performance of peritoneal surface malignancies centers. Surg Oncol Clin N Am (2018) 27(3):507–17. doi: 10.1016/j.soc.2018.02.009 29935686

[71] Kuijpers AM Hauptmann M Aalbers AG Nienhuijs SW de Hingh IH Wiezer MJ. Cytoreduction and hyperthermic intraperitoneal chemotherapy: The learning curve reassessed. Eur J Surg Oncol (2016) 42(2):244–50. doi: 10.1016/j.ejso.2015.08.162 26375923

[72] Chi DS Eisenhauer EL Zivanovic O Sonoda Y Abu-Rustum NR Levine DA. Improved progression-free and overall survival in advanced ovarian cancer as a result of a change in surgical paradigm. Gynecol Oncol (2009) 114(1):26–31. doi: 10.1016/j.ygyno.2009.03.018 19395008

[73] Aletti GD Dowdy SC Gostout BS Jones MB Stanhope CR Wilson TO. Aggressive surgical effort and improved survival in advanced-stage ovarian cancer. Obstet Gynecol (2006) 107(1):77–85. doi: 10.1097/01.AOG.0000192407.04428.bb 16394043

[74] Hall M Savvatis K Nixon K Kyrgiou M Hariharan K Padwick M. Maximal-effort cytoreductive surgery for ovarian cancer patients with a high tumor burden: variations in practice and impact on outcome. Ann Surg Oncol (2019) 26(9):2943–51. doi: 10.1245/s10434-019-07516-3 PMC6682567 31243666

[75] Witkamp AJ de Bree E Van Goethem R Zoetmulder FAN. Rationale and techniques of intra-operative hyperthermic intraperitoneal chemotherapy. Cancer Treat Rev (2001) 27(6):365–74. doi: 10.1053/ctrv.2001.0232 11908929

[76] de Bree E Tsiftsis DD. Principles of perioperative intraperitoneal chemotherapy for peritoneal carcinomatosis. Recent Results Cancer Res (2007) 169:39–51. doi: 10.1007/978-3-540-30760-0_4 17506248

[77] de Bree E Michelakis D Stamatiou D Romanos J Zoras O. Pharmacological principles of intraperitoneal and bidirectional chemotherapy. Pleura Peritoneum (2017) 2(2):47–62. doi: 10.1515/pp-2017-0010 30911633 PMC6405033

[78] Spiliotis J Halkia E de Bree E. Treatment of peritoneal surface malignancies with hyperthermic intraperitoneal chemotherapy-current perspectives. Curr Oncol (2016) 23(3):e266–75. doi: 10.3747/co.23.2831 PMC4900847 27330364

[79] de Bree E Michelakis D. An overview and update of hyperthermic intraperitoneal chemotherapy in ovarian cancer. Expert Opin Pharmacother (2020) 21(12):1479–92. doi: 10.1080/14656566.2020.1766024 32486865

[80] Lim MC Chang SJ Park B Yoo HJ Yoo CW Nam BH. Survival after hyperthermic intraperitoneal chemotherapy and primary or interval cytoreductive surgery in ovarian cancer: A randomized clinical trial. JAMA Surg (2022) 157(5):374–83. doi: 10.1001/jamasurg.2022.0143 PMC8908225 35262624

[81] van Driel WJ Koole SN Sikorska K Schagen van Leeuwen JH Schreuder HWR Hermans RHM. Hyperthermic intraperitoneal chemotherapy in ovarian cancer. N Engl J Med (2018) 378(3):230–40. doi: 10.1056/NEJMoa1708618 29342393

[82] Cascales Campos PA Gonzales Gil A Gil Gomez E Gonzalez Sanchez R Martinez Garcia J Alonso Romero JL. Cytoreductive surgery with or without HIPEC after neoadjuvant chemotherapy in ovarian cancer: A phase 3 clinical trial. Ann Surg Oncol (2022) 29(4):2617–25. doi: 10.1245/s10434-021-11087-7 34812982

[83] Spiliotis J Halkia E Lianos E Kalantzi N Grivas A Efstathiou E. Cytoreductive surgery and HIPEC in recurrent epithelial ovarian cancer: a prospective randomized phase III study. Ann Surg Oncol (2015) 22(5):1570–5. doi: 10.1245/s10434-014-4157-9 25391263

[84] Harter P Reuss A Sehouli J Chiva L du Bois A. Brief report about the role of hyperthermic intraperitoneal chemotherapy in a prospective randomized phase 3 study in recurrent ovarian cancer from spiliotis et al. Int J Gynecol Cancer (2017) 27(2):246–7. doi: 10.1097/IGC.0000000000000864 28114231

[85] Batista TP. Comment on: surgery and HIPEC in recurrent epithelial ovarian cancer: a prospective randomized phase III study. Ann Surg Oncol (2017) 24(Suppl 3):630. doi: 10.1245/s10434-017-6151-5 29094252

[86] Sanz Rubiales Á Del Valle ML. Survival analysis in a randomized trial of HIPEC in ovarian cancer. Ann Surg Oncol (2017) 24(Suppl 3):631. doi: 10.1245/s10434-017-6129-3 29030738

[87] Zivanovic O Chi DS Zhou Q Iasonos A Konner JA Makker V. Secondary cytoreduction and carboplatin hyperthermic intraperitoneal chemotherapy for platinum-sensitive recurrent ovarian cancer: An MSK team ovary phase II study. J Clin Oncol (2021) 39(23):2594–604. doi: 10.1200/JCO.21.00605 PMC8330970 34019431

[88] Shi T Yin S Zhu J Zhang P Liu J Zhu Y. A phase ii trial of cytoreductive surgery combined with niraparib maintenance in platinum-sensitive, secondary recurrent ovarian cancer: SGOG SOC-3 study. J Gynecol Oncol (2020) 31(3):e61. doi: 10.3802/jgo.2020.31.e61 32319233 PMC7189080

